# Preclinical Evaluation of ^99m^Tc-ZHER2:41071, a Second-Generation Affibody-Based HER2-Visualizing Imaging Probe with a Low Renal Uptake

**DOI:** 10.3390/ijms22052770

**Published:** 2021-03-09

**Authors:** Maryam Oroujeni, Sara S. Rinne, Anzhelika Vorobyeva, Annika Loftenius, Joachim Feldwisch, Per Jonasson, Vladimir Chernov, Anna Orlova, Fredrik Y. Frejd, Vladimir Tolmachev

**Affiliations:** 1 Department of Immunology, Genetics and Pathology, Uppsala University, 751 85 Uppsala, Sweden; maryam.oroujeni@igp.uu.se (M.O.); anzhelika.vorobyeva@igp.uu.se (A.V.); fredrik.frejd@affibody.se (F.Y.F.); vladimir.tolmachev@igp.uu.se (V.T.); 2 Department of Medicinal Chemistry, Uppsala University, 751 83 Uppsala, Sweden; sara.rinne@ilk.uu.se; 3 Research Centrum for Oncotheranostics, Research School of Chemistry and Applied Biomedical Sciences, Tomsk Polytechnic University, 634050 Tomsk, Russia; chernov@tnimc.ru; 4 Affibody AB, 171 65 Solna, Sweden; annika.loftenius@affibody.se (A.L.); joachim.feldwisch@affibody.se (J.F.); per.jonasson@affibody.se (P.J.); 5 Nuclear Medicine Department, Cancer Research Institute, Tomsk National Research Medical Center Russian Academy of Sciences, 634009 Tomsk, Russia

**Keywords:** HER2, radionuclide molecular imaging, affibody molecule, technetium-99m, second-generation scaffold

## Abstract

Radionuclide imaging of HER2 expression in tumours may enable stratification of patients with breast, ovarian, and gastroesophageal cancers for HER2-targeting therapies. A first-generation HER2-binding affibody molecule [^99m^Tc]Tc-ZHER2:V2 demonstrated favorable imaging properties in preclinical studies. Thereafter, the affibody scaffold has been extensively modified, which increased its melting point, improved storage stability, and increased hydrophilicity of the surface. In this study, a second-generation affibody molecule (designated ZHER2:41071) with a new improved scaffold has been prepared and characterized. HER2-binding, biodistribution, and tumour-targeting properties of [^99m^Tc]Tc-labelled ZHER2:41071 were investigated. These properties were compared with properties of the first-generation affibody molecules, [^99m^Tc]Tc-ZHER2:V2 and [^99m^Tc]Tc-ZHER2:2395. [^99m^Tc]Tc-ZHER2:41071 bound specifically to HER2 expressing cells with an affinity of 58 ± 2 pM. The renal uptake for [^99m^Tc]Tc-ZHER2:41071 and [^99m^Tc]Tc-ZHER2:V2 was 25–30 fold lower when compared with [^99m^Tc]Tc-ZHER2:2395. The uptake in tumour and kidney for [^99m^Tc]Tc-ZHER2:41071 and [^99m^Tc]Tc-ZHER2:V2 in SKOV-3 xenografts was similar. In conclusion, an extensive re-engineering of the scaffold did not compromise imaging properties of the affibody molecule labelled with ^99m^Tc using a GGGC chelator. The new probe, [^99m^Tc]Tc-ZHER2:41071 provided the best tumour-to-blood ratio compared to HER2-imaging probes for single photon emission computed tomography (SPECT) described in the literature so far. [^99m^Tc]Tc-ZHER2:41071 is a promising candidate for further clinical translation studies.

## 1. Introduction

Human epidermal growth factor receptor type 2 (HER2) is a transmembrane receptor belonging to the receptor tyrosine kinase superfamily. HER2 is overexpressed in a substantial fraction of breast, gastroesophageal, and ovarian cancers, and its elevated expression is associated with a poor prognosis [1,2,3]. HER2-overexpressing breast and gastroesophageal malignant tumors respond to treatment with HER2-targeting monoclonal antibodies, antibody-drug conjugates, and tyrosine kinase inhibitors [3,4]. However, only tumors with high HER2 expression respond to specific targeting therapeutics. Therefore, the information concerning the HER2 expression level is critical for patients’ management [5]. The issue of current methodology for HER2 determination, analysis of biopsy material, and its invasiveness complicates multiple samplings. Thus, the problems of heterogeneous expression of HER2 [6] or an expression change in response to therapy [7] are difficult to solve within this methodology.

**Figure 1:**
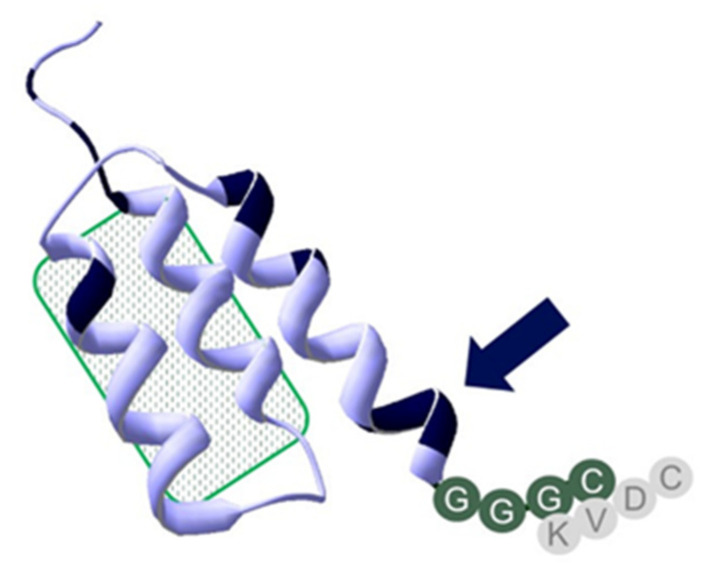
Overview of structural differences, features, and chelator positioning of ZHER2:41071, ZHER2:V2, and ZHER2:2395. All three constructs share the amino acid structure in the binding site, highlighted by the patch with a green pattern. Shared sequences are in a light blue structure, positions with scaffold variation are highlighted in black. The one close to the C-terminal chelator sequence GGGC (marked with an arrow) is consistent of an –SES- amino acids sequence, which replaced an -NDA-sequence to further improve peptide stability and increase hydrophilicity of the affibody molecule. The -SES- motif could have challenged efficient labeling by competing with the GGGC-chelator. Placement of a KVDC chelating sequence within ZHER2:2395 as compared to HER2:V2 and HER2:41071 is shown in gray.

Radionuclide molecular imaging enables non-invasive detection of HER2 in both primary tumors and metastases without false-negative results originating from sampling errors. The first clinical studies proving this approach were performed with the monoclonal antibody trastuzumab labelled with a single photon emitter indium-111 [8,9]. Further progress in the use of antibody-based probes was achieved by the use of a long-lived positron emitter zirconium-89 and application of positron emission tomography (PET), which improved sensitivity and resolution [10]. Still, imaging probes based on therapeutic antibodies clear slowly from blood. This causes a low imaging contrast (even 4–5 days after injection) and an elevated dose burden [11]. The use of small imaging probes, which are based on the VHH domain or engineered scaffold proteins, enables to reduce the optimal imaging time to a few hours after injection and to increase the contrast of imaging, which enhances sensitivity [12]. One of the promising scaffold proteins for radionuclide imaging is the affibody molecule [12,13,14]. Affibody molecules are based on the 58-amino acid cysteine-free scaffold. They can be synthesized chemically or produced in bacteria by the use of recombinant DNA technology [13,14]. A small size, high thermal stability, site-specific radio-labelling, and high affinity are the features rendering affibody molecules excellent targeting agents [13,14]. The first generation affibody molecules have demonstrated an impressive capability to image HER2-expressing tumours in preclinical [15,16,17] and clinical [18] studies. Thereafter, the affibody scaffold was extensively re-engineered and 11 of 45 amino acids in the non-binding part were substituted [19] (Figure 1). The substitutions have improved the yield of peptide synthesis of affibody molecules and their stability. The residual binding to IgG and IgM was essentially eliminated to facilitate the blood clearance. The hydrophilicity of the water-exposed surface was appreciably enhanced by substitution of alanines by serines.

PET studies using the DOTA-conjugated ^68^Ga-labelled second-generation affibody molecule ABY-025 have demonstrated excellent sensitivity and specificity in detecting HER2 expressing metastases in patients with breast cancer [20]. Importantly, a typical clinical investigation using this tracer would give an effective dose in the range of 5–6 mSv [21], which is appreciably lower compared with doses typical for immunoPET (14–22 mSv) [11]. Still, PET imaging is relatively available in Northern America and Western Europe. In South America, Asia, Africa, and even in some European countries, PET imaging is less accessible, while the use of SPECT is more common. SPECT imaging is possible using ^111^In-labelled ABY-025 [22], but this nuclide is expensive and requires medium energy collimators, which decrease resolution and sensitivity.

The use of technetium-99m (T_1/2_ = 6 h) would be much more attractive from a clinical point of view [23]. This nuclide is cheap and readily available because of its generator production. The decay scheme of ^99m^Tc make this radionuclide almost ideal for radiopharmaceutical labelling because of its optimal half-life, benign dosimetry, and possibility to use low-energy high-resolution collimators, which increases the sensitivity of imaging. The labelling of first generation affibody molecules with ^99m^Tc was well investigated. Particularly interesting was the labelling using peptide-based chelators. It was shown that composition of such chelators and their position in affibody molecules might substantially modify biodistribution of ^99m^Tc-labeled affibody molecules and the excretion pathway [24]. A selection of an optimal amino acid sequence of mercaptoacetyl-containing chelators in N-terminus may reduce hepatobiliary excretion [25,26] and uptake in liver and kidneys [27]. More interesting was an application of cysteine-containing peptide-based chelators. It was shown that placement of a cysteine at the C-terminus of affibody molecules forms the N_3_S chelator (-KVDC), which provides stable coupling of ^99m^Tc [16]. The ^99m^Tc-labelled HER2-binding ZHER2:2395 (having a KVDC chelating sequence at the C-terminus) demonstrated an excellent targeting specificity and high imaging contrast of HER2 expression (tumor-to-blood ratio 121 ± 24, 4 h after injection) in a murine model [16]. In this case, the affibody molecule could be produced using both recombinant production and peptide synthesis [28]. An advantage of this approach is that an additional conjugation of a chelator is not required, which simplifies a manufacturing process and reduces production costs. The labelling procedure permits a formulation of freeze-dried kits [29], which facilitates a clinical translation. However, ZHER2:2395 had a high renal uptake (140–190 %ID/g, 4 h after injection) [16]. A strong influence of amino acids’ composition and their order on biodistribution and targeting properties has also been found for this type of chelator [17,30]. The best imaging contrast has been demonstrated by [^99m^Tc]Tc-ZHER2:V2 Affibody molecule containing a –GGGC chelating sequence. It has been shown that the [^99m^Tc](Tc=O)–GGGC complex acts as a non-residualizing label. Activity, which was reabsorbed and rapidly internalized in the kidney, was rapidly excreted. In tumors, where internalization of affibody molecules is slow, the retention of activity was very good due to strong binding of affibody molecules to HER2 on cellular membranes [17]. This provided a combination of a good imaging construct with low renal uptake of [^99m^Tc]Tc-ZHER2:V2.

The –GGGC chelator is a potential candidate for labelling of the second generation of affibody molecules. However, rearranging amino acids of the scaffold might be associated with a risk of creating an additional chelating pocket by clustering amino acids with electron-donating side-chains. It has been suggested that such side-chains might participate in complexing of technetium [31]. This can result in a loss of labelling site-specificity and modification of stability of a conjugate and its pharmacokinetics. For example, introduction of the HEHEHE-tag at the N-terminus of ZHER2:V2 resulted in a noticeable change of biodistribution of the resulting construct, particularly much higher in renal uptake [32]. The binding site composition of the epidermal growth factor receptor (EGFR)--binding affibody molecule ZEGFR:2377 contains a chelating pocket, which competes with –GGGC chelator and makes its use impossible [33]. It was, thus, not a given that the re-engineered HER2-specific affibody scaffold would be compatible with stable labelling with ^99m^Tc using a –GGGC chelating sequence.

The goal of this study was to test the hypothesis that a HER2-binding affibody molecule based on a new scaffold and with a GGGC chelator (designated as ZHER2:41071) is suitable for in vivo imaging. The in vitro and in vivo properties of this variant labelled with ^99m^Tc were compared with the properties of the previously studied anti-ZHER2 affibody molecule conjugates ZHER2:V2 and ZHER2:2395 labelled with ^99m^Tc. In order to prepare for use in human studies, ZHER2:41071 was screened for potential immunogenic epitopes by in silico prediction methodology and compared with ZHER2:V2.

## 2. Results

### 2.1. Production, Purification, and Characterization of Novel Anti-HER2 Affibody Molecules

The peptide synthesis, purification, and lyophilisation performed well for both ZHER2:41071 and ZHER2:V2. The lyophilized molecules were dissolved in PBS with 2 mM EDTA and the purity was determined to be >92% (monomeric form) by reversed phase ltra-performance liquid chromatography (UPLC) and >99% by size exclusion high-performance liquid chromatography (HPLC) for both ZHER2:41071 and ZHER2:V2. The endotoxin levels were low (<0.120 EU/mL). The isoelectric point for both molecules was 7.8.

**Figure 2:**
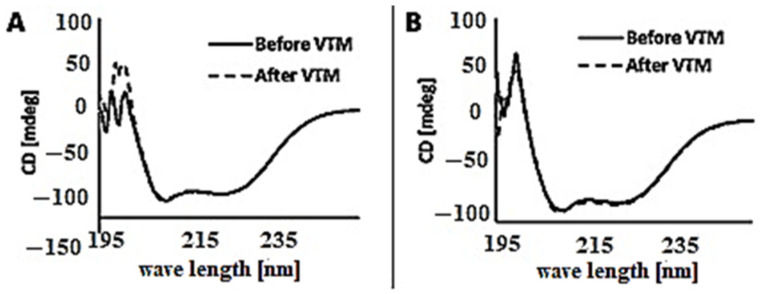
Circular dichroism measurements of (**A**) ZHER2:41071 and (**B**) ZHER:V2 before (black line) and after (dotted line) variable temperature measurements (VTM). The curves indicate alpha-helical conformation and reversibility after heat treatment.

Measurement by circular dichroism showed that the two molecules had a typical alpha helical structure. The overlaid curves from before (black line) and after (dotted line) heat-treatment show that the peptides are reversible after heating to 90 °C (Figure 2).

The melting point (Tm) of ZHER2:41071 and ZHER2:V2 was determined to 62 °C and 68 °C, respectively, by using circular dichroism measurement (Figure S1). No difference in stability was detected between ZHER2:41071 and ZHER2:V2 if stored lyophilized at −20 °C or dissolved in PBS with 2 mM EDTA at −80 and 5 °C up to six months, but long-time storage at 5 °C should be generally avoided. Table S1 shows the results from reversed phase UPLC (the most sensitive chromatography method of the two used).

However, results from UPLC-MS total ion chromatograms (TIC) show that ZHER2:41071 is more stable than ZHER2.V2 after heat treatment to labelling conditions (90 °C for 60 min), i.e., more post-peak variants were developed for ZHER2:V2 (Figures S2 and S3). These variants are most likely de-amidated peptide variants.

The in-silico prediction for potential immunogenic epitopes in the two peptides was evaluated using the IEDB (Immune Epitope Database) web-based MHC II binding prediction tool from National Institute of Health (Bethesda, MD, USA). ZHER2:V2 was predicted to have a higher risk of immunogenicity. The main difference was an epitope predicted at the amino acid residue position 40–48 in ZHER2:V2, which was not found in ZHER2:41071.

### 2.2. Radiolabelling and In Vitro Stability

Data concerning the labelling and stability of ZHER2:2395, ZHER2:41071, and ZHER2:V2 are presented in Table 1. All three variants were successfully labelled with ^99m^Tc, with labelling yields exceeding 95%. Since the radiochemical yield was over 95%, no further purification using NAP-5 was performed for in vitro and in vivo studies. The results of the stability test are presented in Table 1. All conjugates were stable during incubation at 37 °C for 4 h in the presence of excess of phosphate-buffered saline (PBS). Less than 3% release of ^99m^Tc was observed.

**Figure 3:**
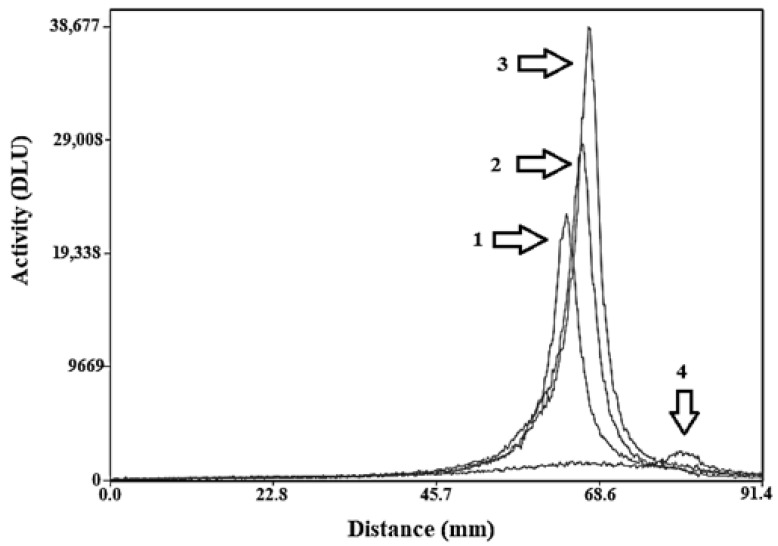
SDS–PAGE analysis of ^99m^Tc-labelled affibody molecules. (1) [^99m^Tc]Tc-ZHER2:41071, (2) [^99m^Tc]Tc-ZHER2:2395, (3) [^99m^Tc]Tc-ZHER2:V2 and (4) ^99m^TcO_4_^−^. The signal was measured as digital light units (DLU) and is proportional to radioactivity at a given point of a lane in the SDS-PAGE gel. Note that the applied activity of ^99m^TcO_4_^−^ was lower compared to applied activity of affibody molecules.

To cross-validate radio-iTLC data (Figure S4), radio-HPLC and SDS-PAGE (sodium dodecyl sulfate–polyacrylamide gel electrophoresis) analysis was performed. The SDS-PAGE analysis (Figure 3) showed no indication of SDS-stable aggregates, decomposition, or release of radionuclide, since no radioactivity bands of higher or lower molecular weight could be observed. According to radio-HPLC (Figure S5), the retention time of all probes was around 8.6–9.2 min. The retention time of the labelled probes (radioactivity detector) was the same as non-labelled ones (ultraviolet detector).

### 2.3. In Vitro Studies

**Figure 4:**
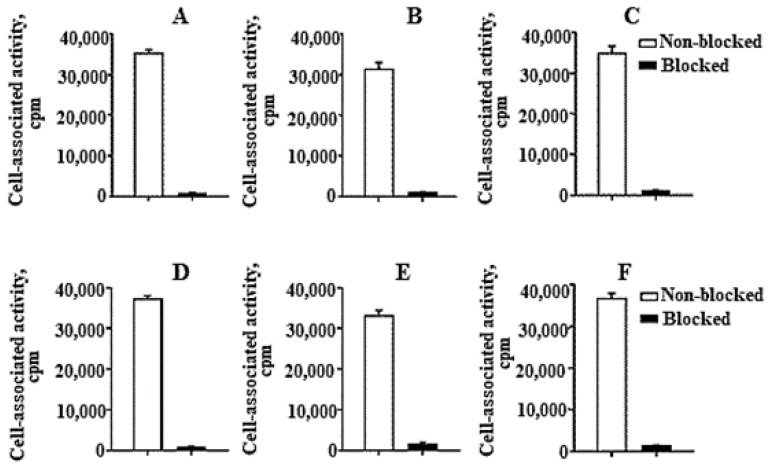
In vitro binding specificity of (**A**,**D**) [^99m^Tc]Tc-ZHER2:41071, (**B**,**E**) [^99m^Tc]Tc-ZHER2:V2 and (**C**,**F**) [^99m^Tc]Tc-ZHER2:2395 to HER2-expressing SKOV3 (**A**–**C**) and BT-474 (**D**–**F**) cell-lines. For the pre-saturation of HER2, a 500-fold molar excess of a non-radioactive affibody molecule was added. The data are presented as an average value from three samples ± SD.

HER2-binding specificity of all three conjugates was tested using a saturation experiment. The binding was significantly (*p* < 5 × 10^−6^) decreased when the cells were pre-saturated with the non-labelled anti-HER2 affibody molecule (Figure 4), demonstrating that the binding was HER2-mediated.

**Figure 5:**
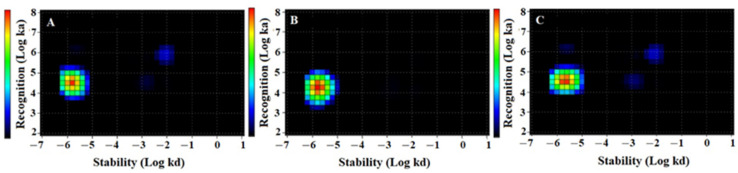
Interaction Map of (**A**) [^99m^Tc]Tc-ZHER2:2395, (**B**) [^99m^Tc]Tc-ZHER2:41071. and (**C**) [^99m^Tc]Tc-ZHER2:V2 binding to HER2-expressing SKOV3 cells. Input data are obtained from LigandTracer measurement of cell-bound activity during association of labelled compounds to and dissociation from SKOV-3 cells. InteractionMap finds individual 1:1 interactions in the input data whose weighted sum explain the observed binding process. The individual interactions are displayed as coloured spots in a ka/kd plot with their colour representing their weight: warmer colours represent more abundant interactions. Binding was measured at three different concentrations: 0.33, 1, and 3 nM. The measurement was performed in duplicates.

The results of the kinetic evaluation of binding and affinity calculations of all radiolabeled conjugates to SKOV3 cells were presented in Figure 5 and Table 2. Representative LigandTracer sensorgrams are presented in Figure S6. According to LigandTracer measurements, the best fit of the binding of all conjugates to the SKOV3 cell line was achieved using a 1:1 model. The Interaction Map calculation showed a rapid association followed by a very slow dissociation for all conjugates, which resulted in picomolar dissociation constants at equilibrium (K_D_). K_D_ values were between 53 and 90 pM.

**Figure 6:**
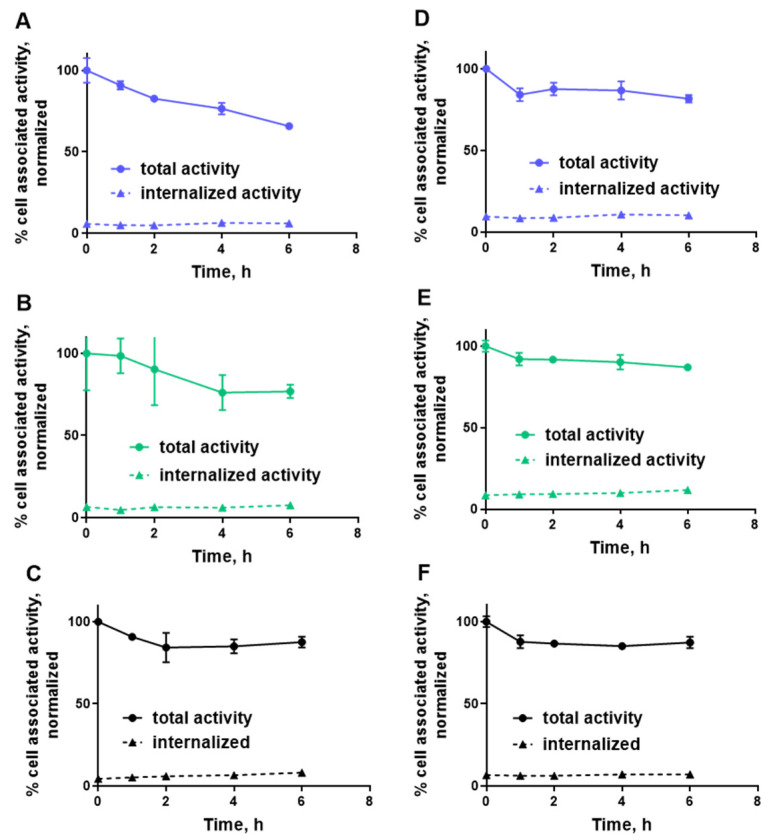
Normalized cellular retention of (**A**,**D**) [^99m^Tc]Tc-ZHER2:41071, (**B**,**E**) [^99m^Tc]Tc-ZHER2:V2 and (**C**,**F**) [^99m^Tc]Tc-ZHER2:2395 to HER2-expressing SKOV3 (**A**–**C**) and BT-474 (**D**–**F**) cell-lines. The data are presented as an average (*n* = 3) and SD. Error bars are not seen because they are smaller than point symbols.

Figure 6 shows the results of cellular retention and an internalization experiment. This experiment showed slow internalization for all conjugates. [^99m^Tc]Tc-ZHER2:2395 showed a tendency to a higher retention after 6 h of incubation (87.6 ± 3.5 and 87.3 ± 3.5% for SKOV3 and BT-474, respectively) than conjugates containing GGGC chelator: [^99m^Tc]Tc- ZHER2:41071 (65.8 ± 2.1 and 81.5 ± 2.3% for SKOV3 and BT-474, respectively) and [^99m^Tc]Tc-ZHER2:V2 (76.8 ± 4.0 and 86.9 ± 1.2%, for SKOV3 and BT-474, respectively). However, this difference was small during the observation period.

### 2.4. In Vivo Studies

**Figure 7:**
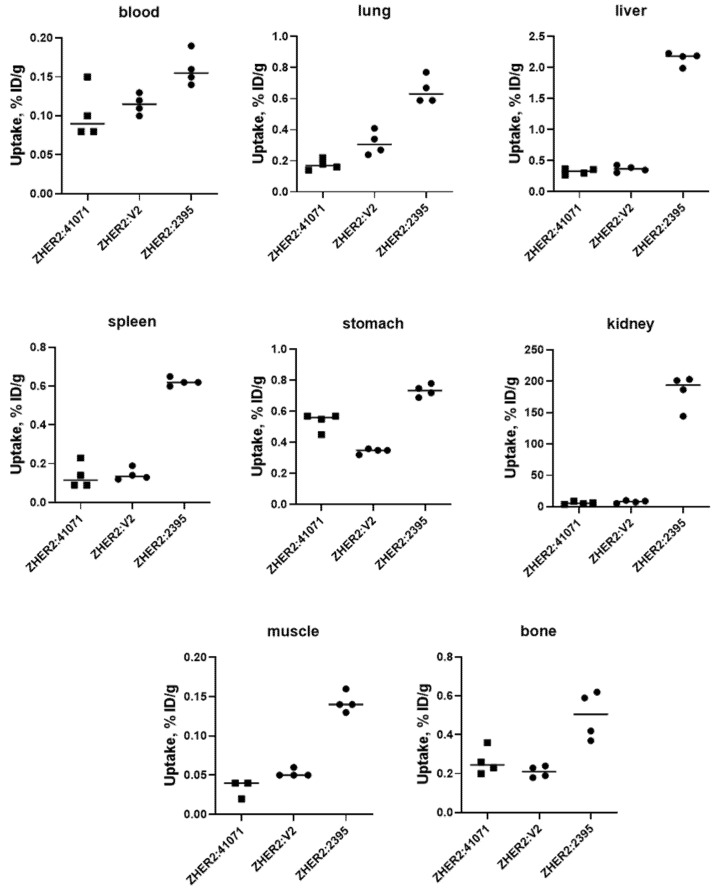
Uptake of ^99m^Tc-labelled affibody molecules in different organs of female NMRI mice at 4 h after injection. Then, 1 µg of labelled affibody molecules (60 kBq, 100 µL in PBS) was injected into the tail vein.

Comparison of [^99m^Tc]Tc-ZHER2:41071, [^99m^Tc]Tc-ZHER2:2395 and [^99m^Tc]Tc-ZHER2:V2 biodistribution in NMRI mice 4 h after injection is presented in Figure 7. Data concerning the biodistribution of [^99m^Tc]Tc-ZHER2:2395 and [^99m^Tc]Tc-ZHER2:V2 were in excellent agreement with the data obtained earlier [17,29]. It is clear that the renal uptake of both GGGC-containing variants (5.9 ± 2.1 and 7.9 ± 2.1 %ID/g for [^99m^Tc]Tc-ZHER2:41071 and [^99m^Tc]Tc- ZHER2:V2, respectively) was significantly lower (*p* < 0.0005) compared with [^99m^Tc]Tc-ZHER2:2395 (183.8 ± 27.3 %ID/g). There was no significant difference of renal uptake between [^99m^Tc]Tc-ZHER2:41071 and [^99m^Tc]Tc-ZHER2:V2. Besides, [^99m^Tc]Tc-ZHER2:41071 and [^99m^Tc]Tc-ZHER2:V2 provided significantly (*p* < 0.05) lower uptakes in lung, liver, spleen, stomach, muscle, and bone than [^99m^Tc]Tc-ZHER2:2395. [^99m^Tc]Tc-ZHER2:41071 has significantly lower blood concentration than [^99m^Tc]Tc-ZHER2:2395.

**Figure 8:**
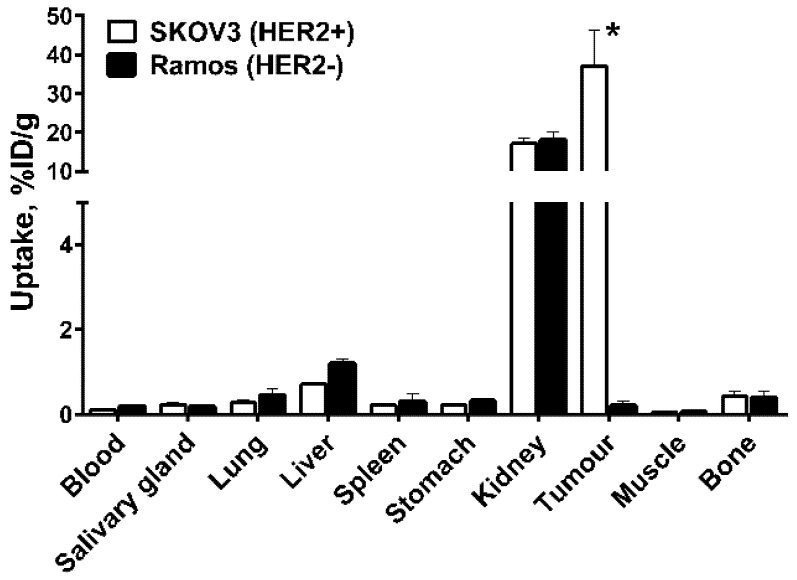
In vivo specificity of [^99m^Tc]Tc-ZHER2:41071 in HER2-negative Ramos xenografts and HER2-positive SKOV3 xenografts at 4 h after injection. The data are presented as the average (*n* = 4) and SD. * *p* < 0.0005.

Experiments in nude mice bearing human cancer xenografts demonstrated that the uptake of [^99m^Tc]Tc-ZHER2:41071 in HER2-expressing SKOV-3 xenografts was significantly (*p* < 0.0005) higher than in HER2-negative Ramos xenografts 4 h after injection (Figure 8), which confirmed that the tumour accumulation was HER2-specific.

Biodistribution data of [^99m^Tc]Tc-ZHER2:41071 at 1, 4, 8, and 24 h after injection are presented in Table 3. [^99m^Tc]Tc-ZHER2:41071 showed efficient targeting, as the tumour uptake amounted to 24 ± 7 %ID/g already at 1 h after injection. The retention of activity in the tumour over time was good for a non-residualizing label. There was no significant difference between tumour uptake 1 h and 8 h after injection, and the tumour uptake decreased only by a factor of two by 24 h after injection. On the opposite side, clearance from normal tissues was rapid. The renal-associated activity was reduced very fast over time (40 ± 1 %ID/g for 1 h p.i. compared to 5.3 ± 0.3 %ID/g for 24 h p.i). Such a biodistribution pattern resulted in very high tumour-to-organ ratios already at 4 h after injection (Table 4).

**Figure 9:**
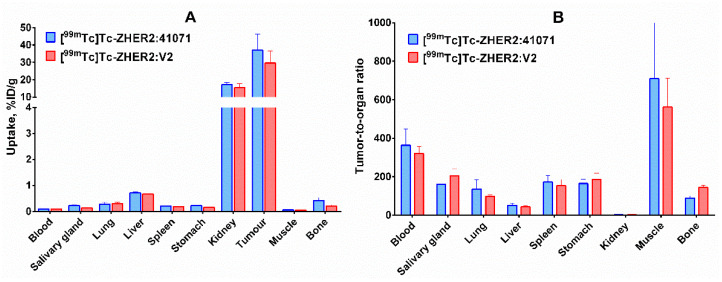
(**A**) Biodistribution and (**B**) tumor-to-organ ratios of selected conjugates, [^99m^Tc]Tc-ZHER2:41071 and [^99m^Tc]Tc-ZHER2:V2, in BALB/C nu/nu mice bearing HER2-expressing SKOV3 xenografts 4 h after injection. The data are presented as the average value (*n* = 4) and SD.

Direct comparison of biodistribution of [^99m^Tc]Tc-ZHER2:41071 and [^99m^Tc]Tc-ZHER2:V2 in the same batch of nude mice bearing human SKOV-3 xenografts 4 h after injection (Figure 9) demonstrated that the biodistribution of tracers with old and new scaffolds is very similar. Thus, the use of a new scaffold did not compromise imaging properties of the affibody molecule. The tumor-to-kidney ratio for both conjugates was approximately on the same level (2.2 ± 0.5 and 1.9 ± 0.2, for [^99m^Tc]Tc-ZHER2:41071 and [^99m^Tc]Tc-ZHER2:V2, respectively).

**Figure 10:**
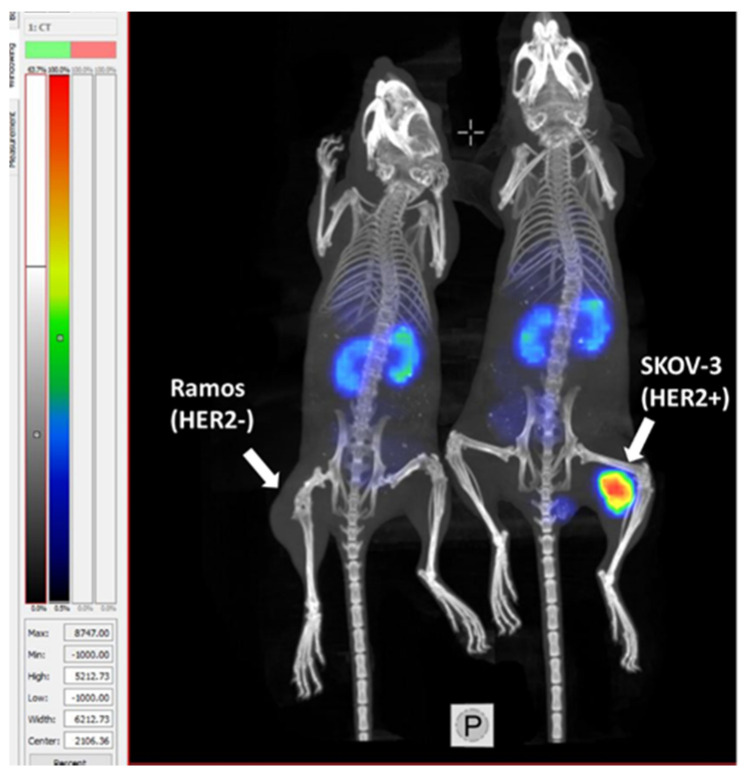
Imaging of HER2-negative Ramos xenograft (left mouse) and HER2-positive SKOV3 xenograft (right mouse) in BALB/C nu/nu mice using [^99m^Tc]Tc- ZHER2:41071. To confirm binding is HER2-specific, one mouse bearing Ramos (HER2-) was used for imaging.

Results of microSPECT/CT imaging (Figure 10) demonstrated that a high-contrast visualization of HER2 expression in HER2-positive tumor using [^99m^Tc]Tc-ZHER2:41071 is feasible. The SKOV3 tumor on right hind leg was clear and, with a high contrast, visualized at 4 h after injection. In agreement with the biodistribution data, the radioactivity uptake in the tumor was considerably higher than in the kidney. Activity uptake in HER2-negative Ramos xenografts was apparently much lower than in the SKOV-3 xenograft.

Estimated absorbed doses of [^99m^Tc]Tc-ZHER2:41071 in humans are presented in Table 5. According to calculations using OLINDA/EXM 1.0, the effective dose should be 0.00066 mSv/MBq (Effective Dose equivalent of 0.00116 mSv/MBq).

## 3. Discussion

Radionuclide molecular imaging has the apparent potential to make targeted cancer treatment more personalized by identifying patients who have tumours with a sufficiently high target expression level. The ^68^Ga-labelled affibody molecule ABY-025 has already demonstrated that the technology can be used for PET imaging of HER2 expression with high specificity and sensitivity [20]. However, PET has limited availability while SPECT imaging is much more available. In addition, the progress in detector engineering and software development makes SPECT/CT an increasingly attractive imaging modality. Modern SPECT/CT cameras provide spatial resolution of about 5.5 mm and quantification accuracy of 5%. Importantly, such cameras remain to be cheaper than PET/CT scanners. Moreover, the use of ^99m^Tc-generators makes the process of radiopharmaceutical production appreciably cheaper, as an installation of a cyclotron is not required. This created a strong motivation for an introduction of affibody-based imaging probes for SPECT into clinics.

Previous preclinical research resulted in development of the [^99m^Tc]Tc-ZHER2:V2 affibody molecules with very attractive imaging properties [17]. However, this tracer utilizes an original affibody scaffold. Apparently, it would be much more appealing to perform a clinical development program using a new, improved scaffold, which provides a better yield in peptide synthesis, higher stability, and lower binding to blood proteins [19]. Therefore, ZHER2:41071, which is a new variant of the HER2-binding affibody molecule, has been designed. It has the same composition of the binding site as ZHER2:V2, but is based on a re-engineered scaffold. In addition, an NDA-sequence close to the C-terminus was replaced by –SES– to further improve peptide stability and increase hydrophilicity of the affibody molecule. The –GGGC chelator was kept at the C-terminus to provide low retention in the kidney (Figure 1). The good in vitro characteristics of ZHER2:V2 were shown to be kept with ZHER2:41071. The two molecules also showed good stability up to six months at −80 °C to 5 °C (Table S1), but ZHER2:41071 was more stable after heat treatment, according to the labelling protocol (90 °C at 60 min) (Figures S1 and S2), which might be due to the replacement of the -NDA-sequence by –SES mentioned above. Re-engineering of the scaffold used for ZHER2:41071 was also shown to reduce the number of potential immunogenic epitopes calculated by a program used for in silico immunogenicity prediction. Although MHCII binding is only one determinant of immunogenicity and might also depend on the particular MHCII allele, such an approach enables us to identify and eliminate potential immunogenicity of protein therapeutics [34]. The modification of the scaffold was very profound, and this could be associated with undesirable changes of properties, such as loss of site specificity of labelling and in negative changes in biodistribution and imaging properties, as discussed in the introduction. Therefore, a clinical translational program would require a new preclinical evaluation of the new improved tracer to make sure that the modifications in the scaffold had no undesirable effects.

In contrast with our concern that a change of several amino acids in the scaffold, especially the introduction of serines, would decrease the labelling efficiency and the beneficial reduction of kidney uptake, and also in contrast from our previous experience with an EGFR-specific affibody molecule where stable labelling could not be achieved with the same chelating sequence [33], it turned out that the effect of the scaffold modifications in this case was minor. The labelling of all tested variants was equally efficient and stable (Table 1). [^99m^Tc]Tc-ZHER2:41071 binding to both HER2-expressing cell lines was highly specific and the specificity was as high as for previously studied HER2-binding affibody molecules (Figure 4). The high affinity of binding to living HER2-expressing cells was preserved (Figure 5 and Table 2) and was even slightly higher than the affinity of [^99m^Tc]Tc-ZHER2:V2. Estimation of the internalization rate is reliable only in the case of a residualizing label, as leakage of radio-metabolites does not lead to underestimation of internalized activity. In vitro data for [^99m^Tc]Tc-ZHER2:2395 (with a residualizing label) demonstrated very low internalized activity for 6 h in both tested cell-lines (Figure 6A,B). Therefore, it is explained that the retention of activity of both variants with non-residualizing labels, [^99m^Tc]Tc-ZHER2:41071 and [^99m^Tc]Tc-ZHER2:V2, was nearly as efficient as the retention of [^99m^Tc]Tc-ZHER2:2395. When the internalization rate is slow, leaking intracellular radio-metabolites make little difference in overall retention, which is mainly determined by strong binding to a membranous protein.

A pilot experiment in normal mice (Figure 7) demonstrated that [^99m^Tc]Tc-ZHER2:41071 and [^99m^Tc]Tc-ZHER2:V2 share multiple biodistribution features. The most conspicuous was the 25–30-fold lower renal uptake of these two affibody molecules compared to [^99m^Tc]Tc-ZHER2:2395. The uptake of [^99m^Tc]Tc-ZHER2:41071 and [^99m^Tc]Tc-ZHER2:V2 in liver was also four-fold lower compared with [^99m^Tc]Tc-ZHER2:2395. This is a favourable feature, as it results in reduced background activity for imaging of liver metastases, which is a frequent challenge in breast cancer. Most likely, the phenomenon of improved biodistribution is caused by the non-residualizing properties of the [^99m^Tc](Tc=O)–GGGC label. The renal reabsorption of affibody molecules causes a rapid internalization and intracellular degradation. Apparently, this process is rapid, and radio-metabolites of non-residualizing labels are eliminated from kidneys by diffusion through lysosomal and cellular membranes [13]. There is always a non-specific binding in normal tissues, which is first and foremost in liver. Analysis of data from previous studies [15,16] shows that affibody molecules with non-residualizing labels have rapid clearance of activity from the liver. It is reasonable to suppose that the hepatic uptake is also associated with quite rapid internalization and lysosomal degradation, leading to a release of non-residualizing labels. Overall, the use of a non-residualizing label resulted in reduced uptake in normal organs and tissues.

Uptake of [^99m^Tc]Tc-ZHER2:41071 in HER2-positive SKOV-3 xenografts was 175-fold higher (*p* < 0.0005) than in HER2-negative Ramos xenografts (Figure 7 and Figure 9), which clearly demonstrates HER2-specificity of targeting. In mice bearing SKOV-3 xenografts, [^99m^Tc]Tc-ZHER2:41071 and [^99m^Tc]Tc-ZHER2:V2 has very similar biodistribution and targeting profiles, and demonstrated similar tumour-to-organ ratios (Figure 9). Thus, a fundamental scaffold modification did not negatively influence the imaging properties of [^99m^Tc]Tc-ZHER2:41071. An evaluation of absorbed doses for humans demonstrated very favourable dosimetry with an effective dose of 0.00066 mSv/MBq (Table 5). For comparison, the effective dose for ^111^In-trastuzumab was 0.19 ± 0.02 mSv/MBq [35], for ^99m^Tc-labelled scaffold protein ADAPT6 0.010 ± 0.003 mSv/MBq [36], and for ^68^Ga-labelled anti-HER2 Affibody molecule ABY-025 0.030 ± 0.003 mSv/MBq [21]. To a high extent, this was determined by low retention of activity in liver and kidneys as well as a short residence time in the whole body.

The comparison of a newly developed imaging probe with the probes developed earlier requires selection of an informative characteristic. Such a characteristic is the imaging contrast provided by a probe. Often, tumour-to-blood ratio is a measure of contrast as blood-born activity contributes to the background. A high imaging contrast is essential for visualization of small metastases, when the partial volume effect results in decreased imaging sensitivity [37]. Therefore, imaging probes with the highest possible imaging contrast are required. There was intense preclinical research aimed at development of SPECT imaging agents based on HER2-targeted therapeutic antibodies trastuzumab [38,39,40] and pertuzumab [41]. Due to bulkiness of intact immunoglobulins (molecular weigh 150 kDa), the tumour-to-blood ratio in murine models was lower than 10 even 72 h after injection. In addition, appreciable unspecific uptake of immunoglobulins in tumours indicated that the imaging specificity might be compromised [38,39]. The low contrast was associated with a low sensitivity in clinics. The maximum contrast of ^111^In-trastuzumab imaging in the clinic was achieved for the first time 168 h after injection [34]. Still, the detection rate of single tumour lesions was 45% [9]. The use of smaller (55 kDa) ^111^In-labelled Fab-fragments of trastuzumab enabled us to reach a tumour-to-blood ratio of 10 at 24 h after injection [42]. Single domain antibodies (sdAb, nanobody) with a molecular weight of 15 kDa were used to develop the smallest immunoglobulin-based HER2-specific imaging probes for SPECT and provided tumour-to-blood ratios in the range of 10–16 1.5 h after injection [43,44].

The use of scaffold proteins permitted us to decrease the size and, thereby, further increase the imaging contrast provided by probes for SPECT visualization of HER2. In mouse models, tumor-to-blood ratios 4 h after injection ranged from 50 to 200 obtained using DARPins [45,46], were around 100 for ADAPTs [46], and ranged from 100–200 for affibody molecules [16,17,29]. [^99m^Tc]Tc-ZHER2:41071 provided the tumour-to-blood ratio of 363 ± 84 in this study, which is the best value provided by a SPECT imaging probe for HER2 so far.

## 4. Materials and Methods

High quality Milli-Q water was used to prepare all buffer solutions. ^99m^Tc was obtained as pertechnetate by elution of an Ultra TechneKow generator (Mallinckrodt, Petten, The Netherlands) with sterile 0.9% sodium chloride (Mallinckrodt, Petten, the Netherlands). The Cyclone Storage Phosphor system (Perkin-Elmer, Wellesley, MA, USA) was used for quantitative measurement of radioactivity distribution in instant, thin-layer, chromatography strips and electrophoresis gels. Radioactivity was measured using an automated gamma-spectrometer with an NaI(TI) detector (1480 Wizard, Wallac, Turku, Finland). For formulation of injection solution, radioactivity was measured using a dose calibrator VDC-405 (Veenstra Instruments BV, Joure, The Netherlands) equipped with an ionization chamber.

In vitro cell studies were performed using the HER2-expressing the ovarian cancer SKOV3 and the breast cancer BT474 cell line, obtained from the American Type Culture Collection (ATCC). Ramos lymphoma cells (ATCC) were used to establish HER2-negative xenografts. Cells were cultured in RPMI medium (Flow Laboratories, Irvine, UK) supplemented with 10% of fetal calf serum, 2 mM of L-glutamine, 100 IU/mL of penicillin, and 100 mg/mL of streptomycin.

Data on in vitro studies and biodistribution were analysed by an unpaired 2-tailed t-test (for comparison of two sets of data) and ANOVA (for comparison of several sets of data) using GraphPad Prism (version 4.00 for Windows, GraphPad Software) to determine significant differences.

### 4.1. Production, Purification, and Characterization of Novel Anti-HER2 Affibody Molecules

ZHER2:41071 and ZHER2:V2 molecules were produced by a solid phase peptide synthesis using the Fmoc/tBu strategy. The molecules were purified by preparative HPLC using a trifluoroacetic acid (TFA)—containing buffer gradient and, finally, the buffer was exchanged to an acetic acid-containing buffer and lyophilized in aliquots of about 1 mg. The lyophilized molecules were stored at −20 °C before characterization and radiolabelling. This work was performed as fee-for-service by the contracting manufacturer Almac Scotland (Penicuik, UK).

The characterization of ZHER2:41071 and ZHER2:V2 was performed to investigate similarities and differences between the molecules before performing the biodistribution studies. The lyophilized molecules were dissolved in PBS containing 2 mM EDTA and filtered through a 0.22-µm filter (Millex GV, Millipore, Burlington, MA, USA) to a concentration of 0.9 mg/mL.

The molecules were characterized by Circular Dichroism (Jasco J-810 spectropolarimeter, Jasco Scandinavia AB, Mölndal, Sweden) for the melting point (Tm) and reversibility of the structure after heating to 90 °C, and with reverse phase ultra-high-performance chromatography (RP-UPLC) (Acquity UPLC CSH-C18, 1.7 µm, 2.1 × 150 mm column, Waters, Milford, MA, USA). Elution by a linear gradient of acetonitrile from 10% to 60% in 0.1% TFA for 24 min at a flow rate of 0.2 mL/min) and size-exclusion high-performance chromatography (SE-HPLC) (Superdex peptide 10/300 GL, Cytiva, Marlborough, MA, USA). Running buffer was 1 × PBS at a flow rate of 0.3 mL/min) for impurity profiles, Isoelectric focusing for isoelectric points (Novex™ pH 3–10 IEF Protein Gels, ThermoFisher Waltham, MA, USA), and endotoxin levels (Endosafe-PTS, Charles River, Wilmington, MA, USA) for safety.

The stability of the lyophilized (at −20 °C) and dissolved molecules (at −80 °C and 5 °C) was also followed for up to six months. RP-UPLC (Acquity UPLC CSH-C18, 1.7 µm, 2.1 × 150 mm column, Waters, Milford, MA, USA), and SE-HPLC (Superdex peptide 10/300 GL, Cytiva Marlborough, MA, USA) were used to evaluate the stability. The stability of the two molecules after treatment at 90 °C for 60 min (radiolabelling conditions) were evaluated by RP-UPLC (Acquity UPLC CSH-C18, 1.7 µm, 2.1 × 100 mm column (Waters, Milford, MA, USA). Gradient 10–50% B-phase over 24 min where the B-phase was 100% acetonitrile +0.1% formic acid. The Flow rate of 0.2 mL/min and in series with a mass spectrometer (Vion-Q-Tof (Waters, Milford, MA, USA)).

Finally, an in silico prediction analysis was performed on ZHER2:41071 to screen for potential immunogenic epitopes in the peptide. ZHER2.V2 was included for comparison. The web-based Immune Epitope Data Base (IEDB, contracted by National Institute of Allergy and Infectious Disease, NIH) MHC II binding prediction tool was used (http://tools.iedb.org/mhcii/, accessed on 3 March 2021).

### 4.2. Radiolabelling and In Vitro Stability

Before labelling, the anti-HER2 affibody molecules was treated with dithiothreitol (DTT, Merck, Darmstadt, Germany) to reduce eventual disulfide bonds formed by cysteine. For this purpose, a solution of DTT (15 µL, 1 M in degassed Milli-Q water) was mixed with affibody molecules (500 µL, 2 mg/mL in degassed PBS). The mixture was incubated at 37 °C for 2 h. Purification of reduced affibody molecules was performed using NAP-5 column equilibrated and eluted with PBS. The solution of the reduced affibody molecules was divided in aliquots, 50 µg in 50 µL-PBS each, and stored at −80 °C.

A freeze-dried labelling kit containing 75 µg of tin (II) chloride dihydrate (Fluka Chemika, Buchs, Switzerland), 5 mg of gluconic acid sodium salt (Celsus Laboratories, Geel, Belgium), and 100 µg of ethylenediaminetetraacetic acid tetra sodium salt (EDTANa_4_) (Sigma-Aldrich, Munich, Germany) was prepared for labelling of affibody molecules with ^99m^Tc, as described earlier [29].

Radiolabelling of all affibody molecules was performed by adding the contents of the freeze-dried kit, dissolved in 100-µL degassed PBS, to 50 µg of the affibody molecule. To the reaction mixture, 100 µL (150–250 MBq) of the generator eluted ^99m^Tc-pertechnetate was added and the vial was degassed to protect the mixture from oxidation. The reaction vial was thoroughly vortexed and incubated at 90 °C for 1 h. Radiochemical yield of affibody molecules were analyzed using instant thin layer chromatography (ITLC-SG) (Agilent Technologies, Santa Clara, CA, USA) developed with PBS (Affibody: Rf = 0.0, other forms of ^99m^Tc: Rf = 1.0). The reduced, hydrolyzed, technetium colloid (RHT) level in the product was measured using pyridine:acetic acid:water (5:3:1.5) as the mobile phase (^99m^Tc colloid: Rf = 0.0, other forms of ^99m^Tc and radio-labelled affibody molecule: Rf = 1.0). Since the radiochemical yield was more than 95% for all conjugates, no further purification was performed. The results of the ITLC measurement were validated by a sodium dodecyl sulphate polyacrylamide gel electrophoresis (SDS-PAGE). This was performed using NuPAGE 4–12% Bis-Tris Gel in2-(N-morpholino)ethanesulfonic acid (MES) buffer (both from Invitrogen AB, Stockholm, Sweden) at 200 V constant for 30 min.

To cross-validate radio-ITLC data further, reverse phase-HPLC conducted on an Elite LaChrom system (Hitachi, VWR, Darmstadt, Germany) consisting of an L-2130 pump, a UV detector (L-2400), and a radiation flow detector (Bioscan, Washington, DC, USA) coupled in series was used. Purity analysis of ^99m^Tc-labelled compounds was performed using an analytical column (Phenomenex, Aschaffenburg, Germany, Luna^®^ 5 µm C18, 100 Å, 4.6 × 150 mm column). HPLC conditions were as follows: A = 10 mM TFA/H_2_O, B = 10 mM TFA/acetonitrile, UV-detection at 220 nm, gradient elution: 0–15 min at 5% to 70% B, 15–18 min at 70% to 95% B, 19–20 min at 5% B, and a flow rate was 1.0 mL/min.

To evaluate stability, fractions of the freshly radio-labelled conjugate (10 µL, 0.4 µg) were incubated with excess amount of PBS (40 µL) for 1 and 4 h at 37 °C. The test was run in triplicates.

### 4.3. In Vitro Studies

For cell studies, the HER2-expressing ovarian carcinoma SKOV-3 and breast carcinoma BT-474 cell lines were used. Cells were seeded cell-culture dishes (35 mm in diameter) with a density of 10^6^ cells/dish. A set of three dishes was used for each data point in vitro binding specificity, cellular retention, and cellular processing studies.

For in vitro binding specificity of each conjugate, cells in three control dishes were pre-saturated with 500-fold excess of non-labelled ZHER2:2395 15 min before addition of the labelled affibody conjugates. The cells in both test and control dishes were incubated with a 0.5-nM labelled conjugate in a humidified incubator (5% CO_2_, 37 °C) for 1 h. The medium was discarded, the cells were washed with cold serum-free medium before trypsin–EDTA solution (0.5 mL per dish) was added, and cells were additionally incubated for 10 min. Detached cells were diluted with 0.5 mL of complete medium, re-suspended, and transferred to fraction tubes. The radioactivity of cells was measured using an automated gamma counter and the cell-bound radioactivity was calculated.

To evaluate the affinity of binding of the radiolabelled conjugates to HER2 receptors, kinetics of binding of ^99m^Tc-labelled affibody molecules to and their dissociation from SKOV3 cells were measured using a LigandTracer Yellow instrument (Ridgeview Instruments AB, Vänge, Sweden), as described earlier [47]. SKOV3 cells were seeded on a local area of a cell culture dish (89 mm in diameter, NunclonTM, NUNC A/S, Roskilde, Denmark). The measurements were performed at room temperature to prevent internalization. Uptake curves were recorded at 0.33, 1, and 3 nM of ^99m^Tc-labelled affibody molecules. Thereafter, the radioactive medium was withdrawn, fresh non-radioactive medium was added, and the dissociation curve was recorded. The data were analysed using the Interaction Map software (Ridgeview Diagnostics AB, Uppsala, Sweden) to calculate the association rate, the dissociation rate, and the dissociation constant at equilibrium (K_D_). The basics of the InteractionMap analysis is described by Altschuh and co-workers [48]. Analysis was performed in duplicates.

Cellular processing after interrupted incubation of cells with radio-labelled conjugates was studied using the methods validated for the affibody molecules earlier [49]. Cells (1 × 10^6^ cells/dish) were incubated with 0.5 nM solution of labelled conjugates at room temperature for 1 h. Thereafter, the medium was removed, the cells were washed, new medium was added, and the cells were placed in a humidified incubator at 37 °C. At 0, 1, 2, 4, and 6 h after incubation, the internalized fraction was determined by an acid wash method. The membrane-bound affibody molecules were removed from cells by treatment with 4 M urea solution in a 0.1 M glycine buffer, pH 2.5, for 5 min on ice. The cell debris containing the internalized conjugates was detached by treatment with 1 M NaOH. Radioactivity of cells was measured, and the percentage of membrane-bound and internalized radioactivity was calculated. The experiments were performed in triplicate.

### 4.4. In Vivo Studies

Animal experiments were performed in accordance with the national legislation for work with laboratory animals. Approval was granted by the Ethical Committee for Animal Research in Uppsala (ethical permission C4/16, decision from 26 February 2016). Groups of four mice per data point were used for these experiments.

To ensure that the scaffold re-engineering did not have a strong adverse effect on in vivo behaviour of [^99m^Tc]Tc-ZHER2:41071, its biodistribution in female NMRI mice 4 h after injection was compared with biodistribution of [^99m^Tc]Tc-ZHER2:V2 and [^99m^Tc]Tc-ZHER2:2395 affibody molecules. Non-tumour bearing mice (weight: 25.5 ± 2.4 g) were injected with 1 µg of ^99m^Tc-labelled affibody molecules (58 ± 1 kBq, 100 µL in PBS) into the tail vein. After 4 h, mice were euthanized by overdosing of of anaesthetic solution (20 μL of solution per gram of body weight: ketamine, 10 mg/mL; Xylazine, 1 mg/mL)This was followed by a heart puncture, and blood samples were collected. Organs and tissue samples were collected and weighed. The organ radioactivity was measured using a gamma-spectrometer along with three standards and empty syringes for each animal. Uptake values for organs were calculated as the percentage-injected dose per gram tissue (%ID/g).

Biodistribution and targeting properties were evaluated in BALB/C nu/nu mice bearing HER2-positive SKOV-3 xenografts. To establish xenografts, 10^7^ SKOV3 cells were subcutaneously injected on the right hind leg of female BALB/c nu/nu mice. As a specificity control, HER2-negative Ramos xenografts were used. 5 × 10^6^ Ramos cells were subcutaneously implanted on the left hind leg of female BALB/c nu/nu in five mice. The experiments were performed two weeks after cell implantation. The average animal weight was 18.2 ± 0.7 g. The average tumour weight was 0.06 ± 0.02 and 0.03 ± 0.01 g for SKOV-3 and Ramos xenografts, respectively. The biodistribution of [^99m^Tc]Tc-ZHER2:41071 was measured 1, 4, 8, and 24 h after injection in mice bearing SKOV-3 xenografts. Three groups of tumour-bearing mice were injected with 4 µg of [^99m^Tc]Tc-ZHER2:41071 (76.5 ± 0.1 kBq, 100 µL in PBS) into the tail vein. For measurement of biodistribution at 24 h after injection, one group of mice was injected with 572 ± 6 kBq, but the injected protein mass was the same, 4 µg. To test HER2-specificity of [^99m^Tc]Tc-ZHER2:41071 accumulation in tumours, one group of animals bearing HER2-negative Ramos xenografts (average mouse weight was 17.6 ± 1.2 g) was injected with 4 µg of [^99m^Tc]Tc-ZHER2:41071 (71.2 ± 0.6 kBq, 100 µL in PBS), and the biodistribution was measured 4 h after injection. For comparison, one group of mice was injected with [^99m^Tc]Tc-ZHER2:V2 (the same activity and dosing), and biodistribution was measured 4 h after injection. The measurement of biodistribution in tumour-bearing mice was performed in the same way as in NMRI mice (see above).

To confirm biodistribution results, a small animal SPECT/CT imaging was performed. One SKOV3 bearing mouse and one Ramos bearing mouse were intravenously injected with 20 MBq/4 µg of [^99m^Tc]Tc-ZHER2:41071. The mice were imaged at 4 h after injection using a nanoScan SPECT/CT scanner (Mediso Medical Imaging Systems, Budapest, Hungary). The mice were euthanized by CO_2_ asphyxiation immediately before being placed in the camera. The computed tomography (CT) acquisition was carried out at the following parameters: energy peak of 50 kV, 670 µA, 480 projections, and 2.29-min acquisition time. SPECT acquisition was performed at the following parameters: ^99m^Tc energy peak of 140 keV, window width of 20%, matrix of 256 × 256, and acquisition time of 1 h. CT images were reconstructed in real-time using Nucline 2.03 Software (Mediso Medical Imaging Systems, Budapest, Hungary). SPECT raw data were reconstructed using Tera-Tomo™ 3D SPECT reconstruction technology.

### 4.5. Dosimetry Evaluation for Humans

To evaluate dosimetry in humans, uptake values in mice were upscaled using the well-established “percent kg/g method”, Equation (1) [50].

(%IA/organ)_human_ = [(%IA/g)_animal_ × (kg_TBweight_)_animal_ × (g_organ_/(kg_TBweight_)_human_] (1)

Organ uptakes for humans were calculated using organ weights of the reference adult female (ICRP publication 23). Residence times were calculated as the area under the curve of exponential fits for human time-activity curves. The remainder of the body residence time was based on radioactivity in carcass. Absorbed doses were estimated using OLINDA/EXM 1.0 software.

## 5. Conclusions

In conclusion, extensive re-engineering of the scaffold did not compromise imaging properties of a HER2-selective affibody molecule labelled with ^99m^Tc using a –GGGC chelator. The new probe, [^99m^Tc]Tc-ZHER2:41071 provided the best tumour-to-blood ratio compared to the probes described in the research. [^99m^Tc]Tc-ZHER2:41071 is a promising candidate for further clinical translation studies.

## Figures and Tables

**Table 1:** Radiolabelling of affibody molecules with ^99m^Tc and stability of the conjugate.

Compound	Radio-Labelling Yield, %	Stability in PBS, %	
		1 h	4 h
ZHER2:2395	98.6 ± 1.4	96.2 ± 0.8	97.1 ± 0.4
ZHER2:41071	95.6 ± 2.7	98.7 ± 0.7	97.5 ± 0.9
ZHER2:V2	96.3 ± 1.9	98.1 ± 0.2	97.2 ± 0.3

**Table 2:** Apparent equilibrium dissociation (K_D_) constants for the interaction between [^99m^Tc]Tc-labelled affibody molecules and HER2-expressing SKOV3 cells determined using an Interaction Map analysis of the LigandTracer sensorgrams.

Label	k_a_ (1/M×s) × 10^4^	k_d_ (1/s) × 10^−6^	K_D_ (pM)
[^99m^Tc]Tc-ZHER2:2395	2.9 ± 0.3	1.50 ± 0.05	53 ± 4
[^99m^Tc]Tc-ZHER2:41071	3.7 ± 0.1	2.17 ± 0.05	58 ± 2
[^99m^Tc]Tc-ZHER2:V2	2.4 ± 0.8	2.0 ± 0.1	90 ± 22

**Table 3:** Biodistribution of [^99m^Tc]Tc-ZHER2:41071 in BALB/C nu/nu mice bearing SKOV3 xenografts. Data are expressed as %ID/g and are averages from four mice ± SD.

Site	Uptake, %ID/g			
	1 h	4 h	8 h	24 h
Blood	1.6 ± 0.2	0.102 ± 0.004	0.08 ± 0.03	0.06 ± 0.02
Salivary gland	0.8 ± 0.1	0.23 ± 0.05	0.07 ± 0.01	0.06 ± 0.02
Lung	2.7 ± 0.6	0.28 ± 0.07	0.14 ± 0.01	0.11 ± 0.02
Liver	2.0 ± 0.4	0.71 ± 0.04	0.48 ± 0.08	0.42 ± 0.05
Spleen	0.9 ± 0.1	0.21 ± 0.02	0.17 ± 0.06	0.18 ± 0.02
Stomach	1.4 ± 0.3	0.22 ± 0.03	0.13 ± 0.03	0.11 ± 0.03
Kidney	40 ± 1	17 ± 1	9 ± 1	5.3 ± 0.3
Tumour	24 ± 7	37 ± 9	23 ± 6	13 ± 2
Muscle	0.5 ± 0.1	0.06 ± 0.02	0.04 ± 0.02	0.03 ± 0.02
Bone	1.4 ± 0.4	0.4 ± 0.1	0.21 ± 0.04	0.27 ± 0.02
Uterus	1.8 ± 0.5	0.20 ± 0.08	0.08 ± 0.04	0.10 ± 0.03
Brain	0.06 ± 0.01	0.0110 ± 0.0005	0.009 ± 0.002	0.009 ± 0.003
Pancreas	0.48 ± 0.01	0.054 ± 0.001	0.05 ± 0.01	0.05 ± 0.01
Small Intestine	1.2 ± 0.3	0.13 ± 0.07	0.12 ± 0.03	0.08 ± 0.02

**Table 4:** Tumour-to-organ ratios for [^99m^Tc]Tc-ZHER2:41071 in BALB/C nu/nu mice bearing SKOV3 xenografts. Data are expressed as %ID/g and are averages from four mice ± SD.

Site	Tumour-to-Organ Ratio			
	1 h	4 h	8 h	24 h
Blood	16 ± 6	363 ± 84	284 ± 85	250 ± 9
Salivary gland	29 ± 6	161 ± 27	325 ± 118	220 ± 53
Lung	9 ± 2	136 ± 49	170 ± 51	127 ± 20
Liver	12 ± 1	52 ± 11	50 ± 20	32 ± 3
Spleen	27 ± 7	172 ± 34	148 ± 66	75 ± 10
Stomach	18 ± 5	165 ± 23	177 ± 38	138 ± 14
Kidney	0.6 ± 0.2	2.1 ± 0.5	2.8 ± 0.9	2.4 ± 0.4
Muscle	52 ± 8	709 ± 300	527 ± 95	538 ± 324
Bone	18 ± 1	89 ± 12	115 ± 46	48 ± 9
Uterus	14 ± 4	232 ± 178	315 ± 233	137 ± 37
Brain	423 ± 155	3397 ± 947	2629 ± 947	1548 ± 684
Pancreas	51 ± 15	690 ± 175	477 ± 193	276 ± 40
Small Intestine	21 ± 4	288 ± 103	209 ± 127	166 ± 49

**Table 5:** Calculated absorbed dose (mGy/MBq) for [^99m^Tc]Tc-ZHER2:41071 in humans using OLINDA/EXM 1.0.

Organ	Absorbed Dose (mGy/MBq)	Organ	Absorbed Dose (mGy/MBq)
Adrenals	0.0009	Muscle	0.00034
Brain	0.00016	Ovaries	0.00042
Breasts	0.00029	Pancreas	0.00073
Gallbladder wall	0.0007	Red marrow	0.00062
Lower large intestine wall	0.0004	Osteogenic cells	0.0026
Small intestine	0.00049	Skin	0.00021
Stomach wall	0.00052	Spleen	0.00078
Upper large intestine wall	0.0005	Thymus	0.00049
Heart wall	0.00160	Thyroid	0.00029
Kidney	0.0069	Urinary bladder wall	0.00037
Liver	0.00088	Uterus	0.00051
Lungs	0.0007	Total body	0.00048

## Data Availability

Data is contained within the article or supplementary material.

## Supplementary Materials

The following are available online at https://www.mdpi.com/1422-0067/22/5/2770/s1, Figure S1. Variable temperature measurement (VTM) on ZHER2:41071 and ZHER2.V2. At the VTM, the absorbance was measured at 221 nm while the temperature was raised from 20 to 90 °C with a temperature slope of 5 °C/min. Tm is the temperature where the slope of the CD vs. temperature curve is the highest. Figure S2: ZHER2:41071 Affibody peptide stability at labelling conditions. Full size (A) and enlarged total ion chromatogram (B) before (grey) and after (black) heat treatment. Figure S3. ZHER2:V2 Affibody peptide stability at labelling conditions. Full size (A) and enlarged total ion chromatogram (B) before (grey) and after (black) heat treatment. Figure S4. Distribution of radioactivity of (A) [^99m^Tc]Tc-ZHER2:2395, (B) [^99m^Tc]Tc-ZHER2:41071 and (C) [^99m^Tc]Tc-ZHER2:V2 along the ITLC strip. The retardation factor of labelled conjugate is 0.0 and that of free 99mTc is 1.0. Figure S5. Characterization of anti-HER:2 affibody molecules. Reversed-phase HPLC chromatograms of non-labelled (A) ZHER2:2395, (C) ZHER2:41071, and (E) ZHER2:V2, and the radio-chromatogram of (B) [^99m^Tc]Tc-ZHER2:2395, (D) [^99m^Tc]Tc-ZHER2:41071 and (F) [^99m^Tc]Tc-ZHER2:V2. The retention times (Rt) are expressed in minutes. Figure S6. Binding curve of (A) [^99m^Tc]Tc-ZHER2:2395, (B) [^99m^Tc]Tc-ZHER2:41071, and (C) [^99m^Tc]Tc-ZHER2:V2 binding to HER2-expressing SKOV3 cells. Input data are obtained from the LigandTracer measurement of cell-bound activity during the association of labelled compounds to and dissociation from SKOV-3 cells. Binding was measured at three different concentrations: 0.33, 1, and 3 nM. Measurement was performed in duplicates. Table S1. Storage stability of ZHER2:41071 and ZHER2:V2 by Reversed Phase UPLC.
