# Supplementary material for: Preclinical Evaluation of 99mTc-ZHER2:41071, a Second-Generation Affibody-Based HER2-Visualizing Imaging Probe with a Low Renal Uptake
Source: Int J Mol Sci. 2021 Mar 9;22(5):2770. doi: 10.3390/ijms22052770 (PMC7967187; doi:10.3390/ijms22052770)
Supplement: Supplementary file 1 [file ijms-22-02770-s001.pdf]

Table S1. Storage stability of ZHER2:41071 and ZHER2:V2 by Reversed Phase UPLC.

Peptide	Storage Conditions	% Monomer	% Dimer	% Unidentified Peaks
ZHER2:41071 Lyophilized	−0 °C, 0 month	92.9	2.3	4.8
	−20 °C, 6 months	91.8	2.6	5.6
ZHER2:41071 Dissolved *	−80 °C, 1 month	91.1	4.1	4.8
	−80 °C, 3 months	90.2	4.2	5.6
	−80 °C, 6 months	90.9	3.9	5.2
	+5 °C, 1 month	88.4	6.5	5.1
	+5 °C, 3 months	84.8	9.3	5.9
	+5 °C, 6 months	81.8	11.5	6.7
ZHER2:V2 Lyophilized	−20 °C, 0 month	N/A	N/A	N/A
	−20 °C, 6 months	92.8	2.5	4.7
ZHER2:V2 Dissolved*	−80 °C, 1 month	90.5	4.5	5.0
	−80 °C, 3 months	92.0	5.0	3.0
	−80 °C, 6 months	91.5	4.9	3.6
	+5 °C, 1 month	88.8	6.6	4.7
	+5 °C, 3 months	88.2	8.3	3.5
	+5 °C, 6 months	85.8	9.9	4.3

* Dissolved in PBS pH 7.4 containing 2 mM EDTA.

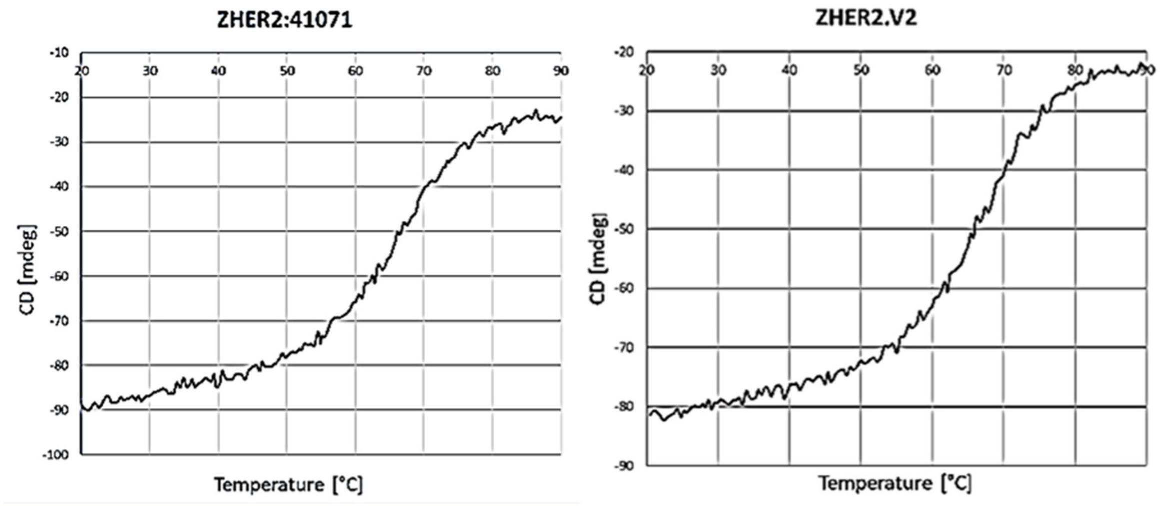

Figure S1. Variable temperature measurement (VTM) on ZHER2:41071 and ZHER2.V2. At the VTM the absorbance was measured at 221 nm while the temperature was raised from 20 to 90 °C with a temperature slope of 5 °C/min. Tm is the temperature where the slope of the CD vs temperature curve is the highest.

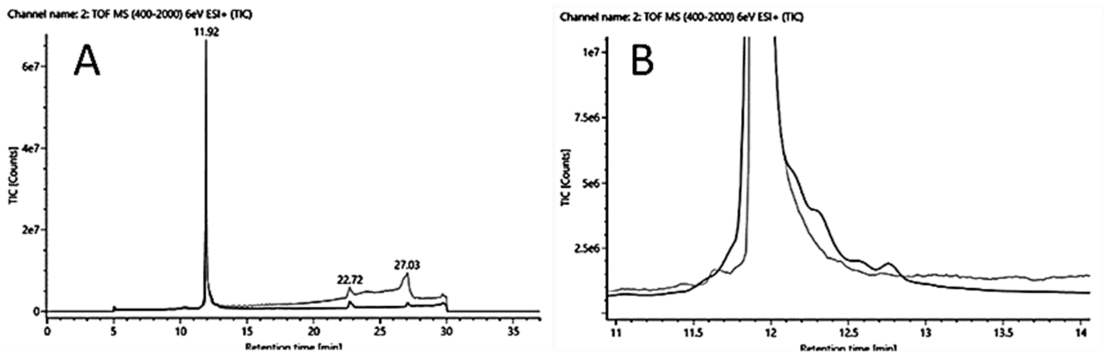

Figure S2. ZHER2:41071 Affibody peptide stability at labelling conditions. Full size (A) and enlarged total ion chromatogram (B) before (grey) and after (black) heat treatment.

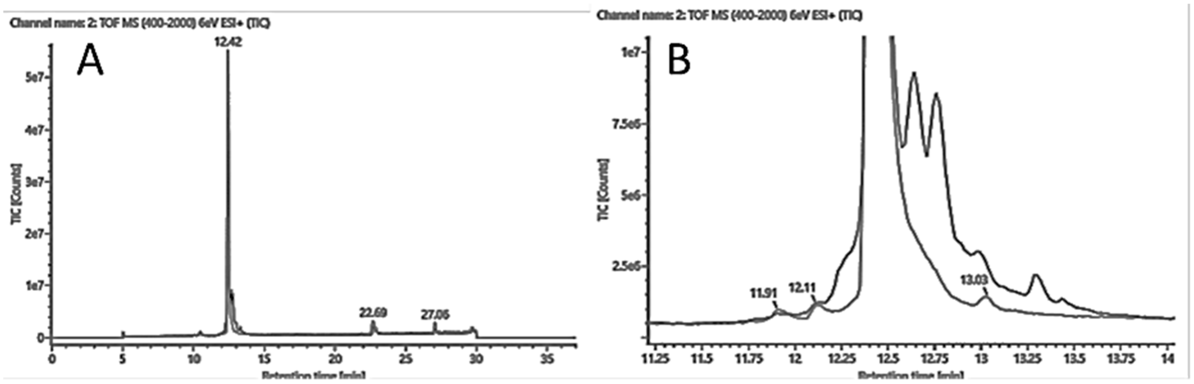

Figure S3. ZHER2:V2 Affibody peptide stability at labelling conditions. Full size (A) and enlarged total ion chromatogram (B) before (grey) and after (black) heat treatment.

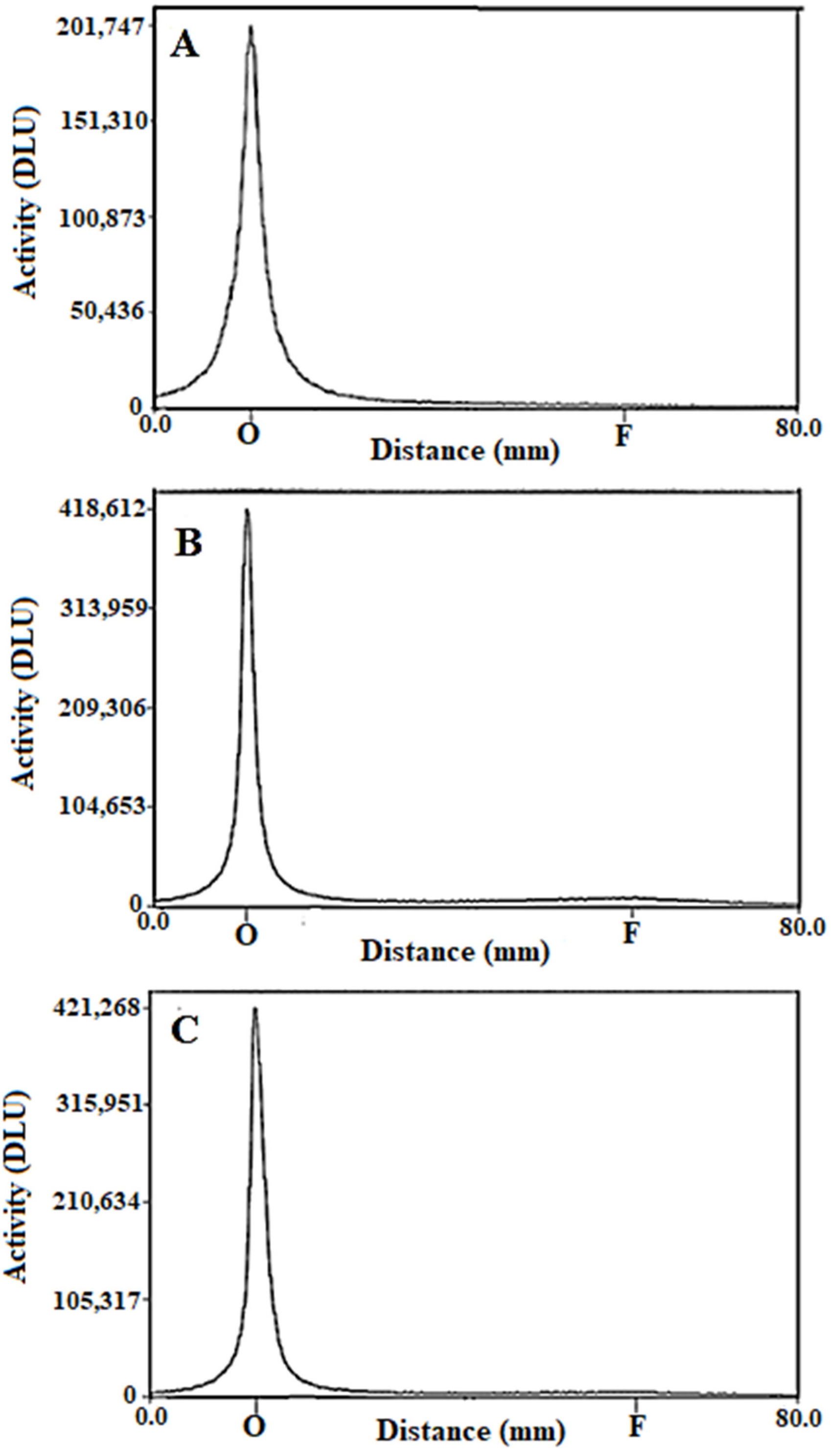

Figure S4. Distribution of radioactivity of (A) [^{99m}Tc]Tc-ZHER2:2395, (B) [^{99m}Tc]Tc- ZHER2:41071 and (C) [^{99m}Tc]Tc-ZHER2:V2 along ITLC strip. Retardation factor of labelled conjugate is 0.0 and that of free ^{99m}Tc is 1.0.

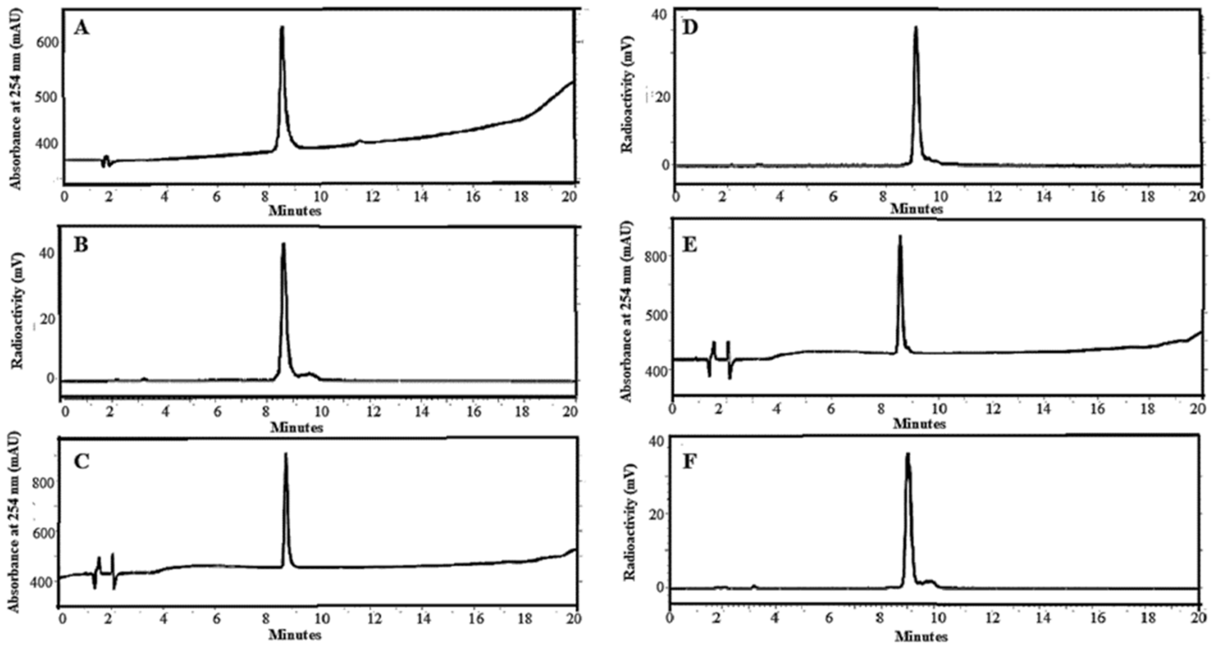

Figure S5. Characterization of anti-HER:2 Affibody molecules. Reversed-phase HPLC chromatograms of non-labelled (A) ZHER2:2395, (C) ZHER2:41071 and (E) ZHER2:V2; and the radiochromatogram of (B) [^{99m}Tc]Tc-ZHER2:2395, (D) [^{99m}Tc]Tc-ZHER2:41071 and (F) [^{99m}Tc]Tc-ZHER2:V2.The retention times (Rt) are expressed in minutes.

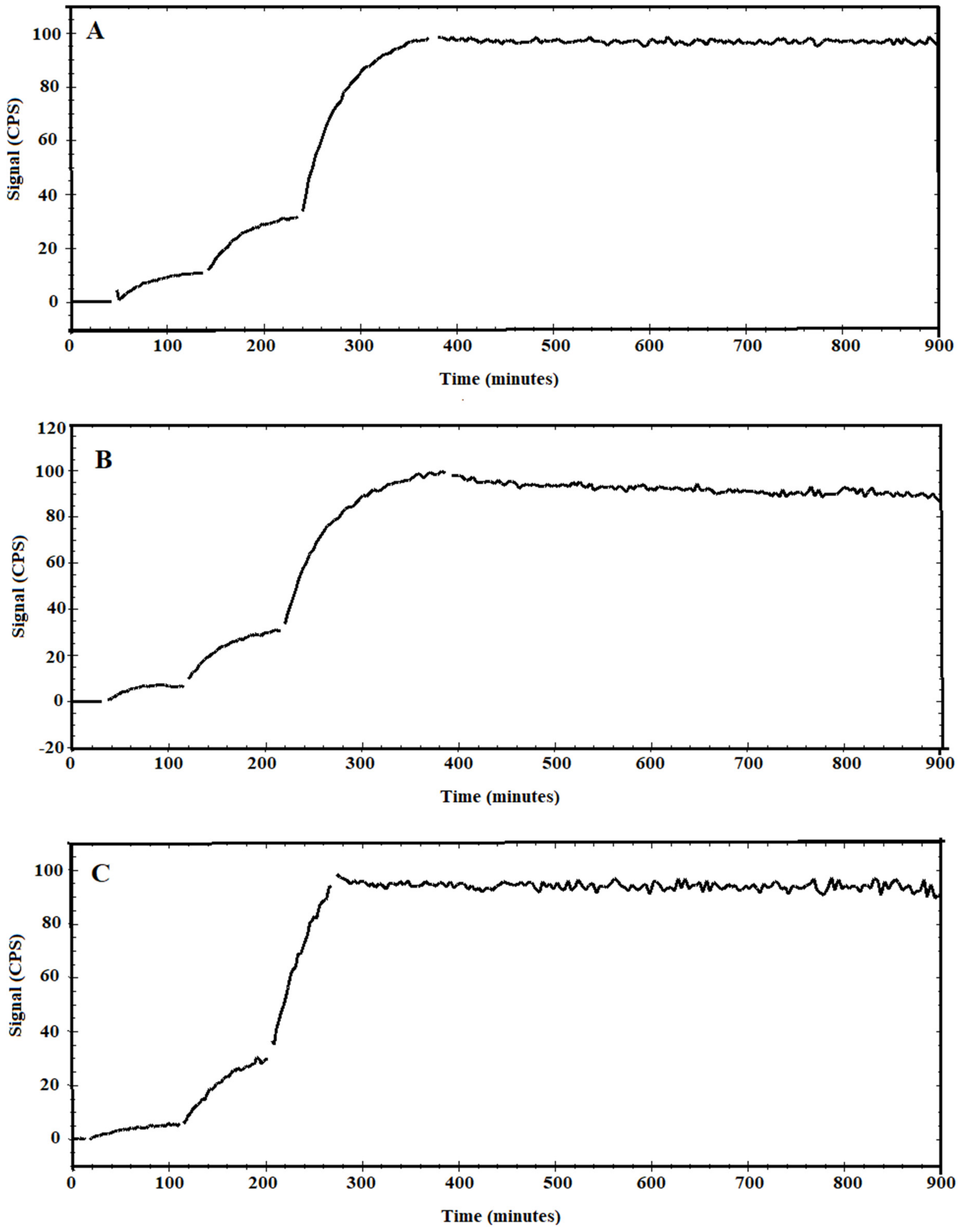

Figure S6. Binding curve of (A) [^{99m}Tc]Tc-ZHER2:2395, (B) [^{99m}Tc]Tc-ZHER2:41071 and (C) [^{99m}Tc]Tc-ZHER2:V2 binding to HER2-expressing SKOV3 cells. Input data are obtained from LigandTracer measurement of cell-bound activity during association of labelled compounds to and dissociation from SKOV-3 cells. Binding was measured at three different concentrations: 0.33, 1 and 3 nM. Measurement was performed in duplicates.
